# Quantification of strain and charge co-mediated magnetoelectric coupling on ultra-thin Permalloy/PMN-PT interface

**DOI:** 10.1038/srep03688

**Published:** 2014-01-14

**Authors:** Tianxiang Nan, Ziyao Zhou, Ming Liu, Xi Yang, Yuan Gao, Badih A. Assaf, Hwaider Lin, Siddharth Velu, Xinjun Wang, Haosu Luo, Jimmy Chen, Saad Akhtar, Edward Hu, Rohit Rajiv, Kavin Krishnan, Shalini Sreedhar, Don Heiman, Brandon M. Howe, Gail J. Brown, Nian X. Sun

**Affiliations:** 1 Department of Electrical and Computer Engineering, Northeastern University, Boston, MA, USA; 2 Materials and Manufacturing Directorate, Air Force Research Laboratory, Wright-Patterson AFB, OH, USA; 3 Department of Physics, Northeastern University, Boston, MA; 4 Shanghai Institute of Ceramics, Chinese Academy of Sciences, Shanghai, China; 5 Winchester High School, Winchester, MA; 6 Foxborough High School, Foxborough, MA; 7 Boston Latin School, Boston, MA; 8 Phillips Exeter Academy, Exeter, NH; 9 Advanced Math & Science Academy Charter School, Marlborough MA; 10 Weston High School, Weston, MA; 11 These authors contributed equally to this work.

## Abstract

Strain and charge co-mediated magnetoelectric coupling are expected in ultra-thin ferromagnetic/ferroelectric multiferroic heterostructures, which could lead to significantly enhanced magnetoelectric coupling. It is however challenging to observe the combined strain charge mediated magnetoelectric coupling, and difficult in quantitatively distinguish these two magnetoelectric coupling mechanisms. We demonstrated in this work, the quantification of the coexistence of strain and surface charge mediated magnetoelectric coupling on ultra-thin Ni_0.79_Fe_0.21_/PMN-PT interface by using a Ni_0.79_Fe_0.21_/Cu/PMN-PT heterostructure with only strain-mediated magnetoelectric coupling as a control. The NiFe/PMN-PT heterostructure exhibited a high voltage induced effective magnetic field change of 375 Oe enhanced by the surface charge at the PMN-PT interface. Without the enhancement of the charge-mediated magnetoelectric effect by inserting a Cu layer at the PMN-PT interface, the electric field modification of effective magnetic field was 202 Oe. By distinguishing the magnetoelectric coupling mechanisms, a pure surface charge modification of magnetism shows a strong correlation to polarization of PMN-PT. A non-volatile effective magnetic field change of 104 Oe was observed at zero electric field originates from the different remnant polarization state of PMN-PT. The strain and charge co-mediated magnetoelectric coupling in ultra-thin magnetic/ferroelectric heterostructures could lead to power efficient and non-volatile magnetoelectric devices with enhanced magnetoelectric coupling.

Artificial multiferroic heterostructures with combined ferroelectric and ferromagnetic layers have attracted an ever-increasing amount of interest recently due to their strong magnetoelectric coupling at room temperature^1^,^2^,^3^,^4^,^5^,^6^. The magnetoelectric coupling leads to voltage control of magnetism, or magnetic field manipulation of polarization, which enables low power consumption, fast dynamic response, low loss and compact devices^6^,^7^. Those devices include magnetoelectric sensors^8^,^9^,^10^, voltage tunable RF/microwave magnetic devices^11^,^12^,^13^,^14^ and spintronics^15^,^16^,^17^,^18^,^19^, which show great potential in real applications. A lot of efforts have been devoted to achieving strong magnetoelectric coupling, in particular, the modulation of magnetic anisotropy by piezoelectric strain mediated magnetoelectric coupling^20^,^21^,^22^. However the strain-mediated magnetoelectric coupling is constrained by the substrate clamping effect which can diminish the magnetoelectric coupling in thin film multiferroic heterostructures.

Strong magnetoelectric coupling has been demonstrated in magnetic/dielectric or magnetic/ferroelectric thin film heterostructures through a voltage controllable magnetic surface anisotropy mediated by spin polarized charge^23^,^24^,^25^,^26^,^27^,^28^,^29^. Combined strain-mediated and charge-mediated magnetoelectric coupling is expected in ultra-thin magnetic film/ferroelectric slabs, which has the potential for achieving even stronger magnetoelectric coupling. For example, a multiferroic heterostructure with a magnetic semiconductor, 4 nm La_0.8_Sr_0.2_MnO_3_, on PZT produced a hysteretic-like M-E curve at 100 K due to a charge mediated magnetoelectric coupling^24^,^30^; while a characteristic strain-mediated piezoelectric “butterfly” like M-E curve was observed in a heterostructure with 50 nm La_0.7_Sr_0.3_MnO_3_ on PMN-PT^31^. Shu et al. reported a thickness-dependent M-E behavior in Ni/BTO multiferroic heterostructures through the voltage controlled magneto-optical Kerr signal, where the charge-mediated magnetic surface anisotropy increasingly dominates over the magnetoelastic anisotropy when decreasing the thickness of Ni thin film down to 5 nm^32^. It is however difficult to separate the strain mediated magnetic coupling from the charge mediated magnetic coupling in such ultra-thin magnetic films on ferroelectric substrates. This is due to the weak magnetic signal that results from the ultra-thin film; and there has been no report of the precise measurements of the strain and charge co-mediated magnetoelectric coupling.

In this work, a precise quantification of the ferromagnetic resonance of multiferroic heterostructures was carried out in order to distinguish the strain-mediated and charge-mediated magnetoelectric coupling. First, an ultra-thin ferromagnetic permalloy film of 1 nm was deposited on a (011) oriented PMN-PT (0.71 Pb(Mg_1/3_ Nb_2/3_)O_3_-0.29 PbTiO_3_) single crystal substrate. Measurement of the voltage controled ferromagnetic resonance of the ultra-thin NiFe/PMN-PT multiferroic heterostructures in an electron spin resonance (ESR) spectrometer demonstrated a strong combined strain and charge-mediated magnetoelectric coupling. As a comparison, a 5 nm Cu film was inserted in between the NiFe and PMN-PT layers, the NiFe/Cu/PMN-PT heterostructure presented only strain-mediated magnetoelectric coupling. By distinguishing the strain and surface charge magnetoelectric effect strength, we obtained the hysteretic loop like M-E curve, in which the voltage controlled switch of the magnetization corresponds to the switch of the ferroelectric polarization.

## Results

**Figure 1:**
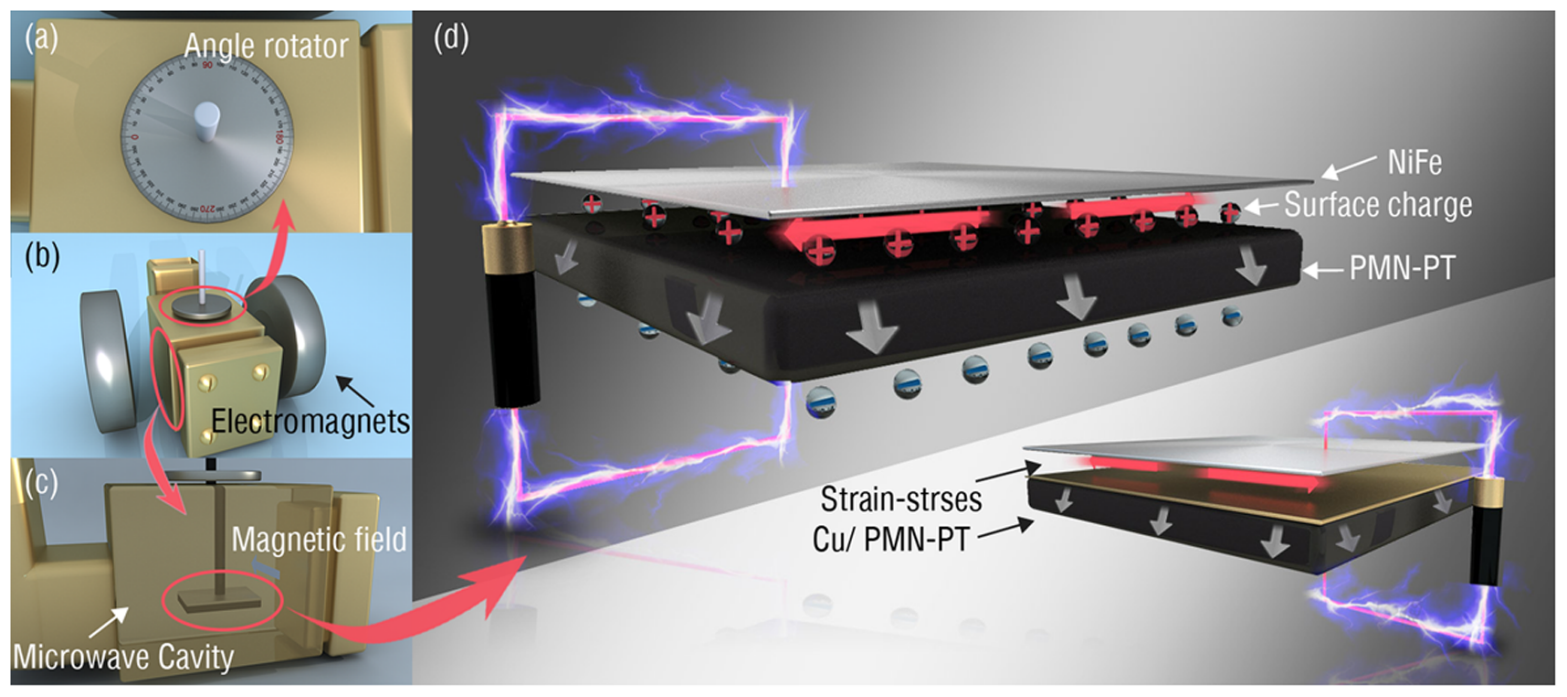
(a) Schematic of electron spin resonance (ESR) system with angle rotator designed for angular dependence FMR field measurement in top view; (b) side view of the whole structure of the ESR system; (c) schematic of the ESR system with NiFe/PMN-PT sample placed in microwave cavity for FMR field sweeping mode (not to scale). (d) NiFe/PMN-PT and NiFe/Cu/PMN-PT multiferroic heterostructures with applied electric field to induce surface charge and strain across the interface.

For the NiFe/PMN-PT multiferroic heterostructure, an ultra-thin (1 nm) ferromagnetic film of Ni_0.79_Fe_0.21_ was deposited on a (011)-PMN-PT single crystal using magnetron sputtering, with a 5 nm Cu film as a top cap on NiFe. The thickness of the films was precisely controlled by deposition time. For the control sample, a Cu layer was inserted between the NiFe and PMN-PT substrate to create a Cu(5 nm)/NiFe(1 nm)/Cu(5 nm)/PMN-PT heterostructure. The NiFe alloy was selected due to its low magnetostriction constant (~10 ppm) and small ferromagnetic resonance (FMR) linewidth. The magnetization of NiFe/PMN-PT and NiFe/Cu/PMN-PT heterostructures were measured to be around 875 emu cm^−3^ (1.1 T) using a SQUID magnetometer as shown in Figure S2. Both samples were characterized by the electron spin resonance (ESR) method for precisely quantifying the magnetic anisotropy induced by the applied electric field, as schematically shown in Figure 1. The FMR was measured in field sweeping mode at 9.5 GHz. Polarization of the ferroelectric PMN-PT substrate was shown in Figure S1.

**Figure 2:**
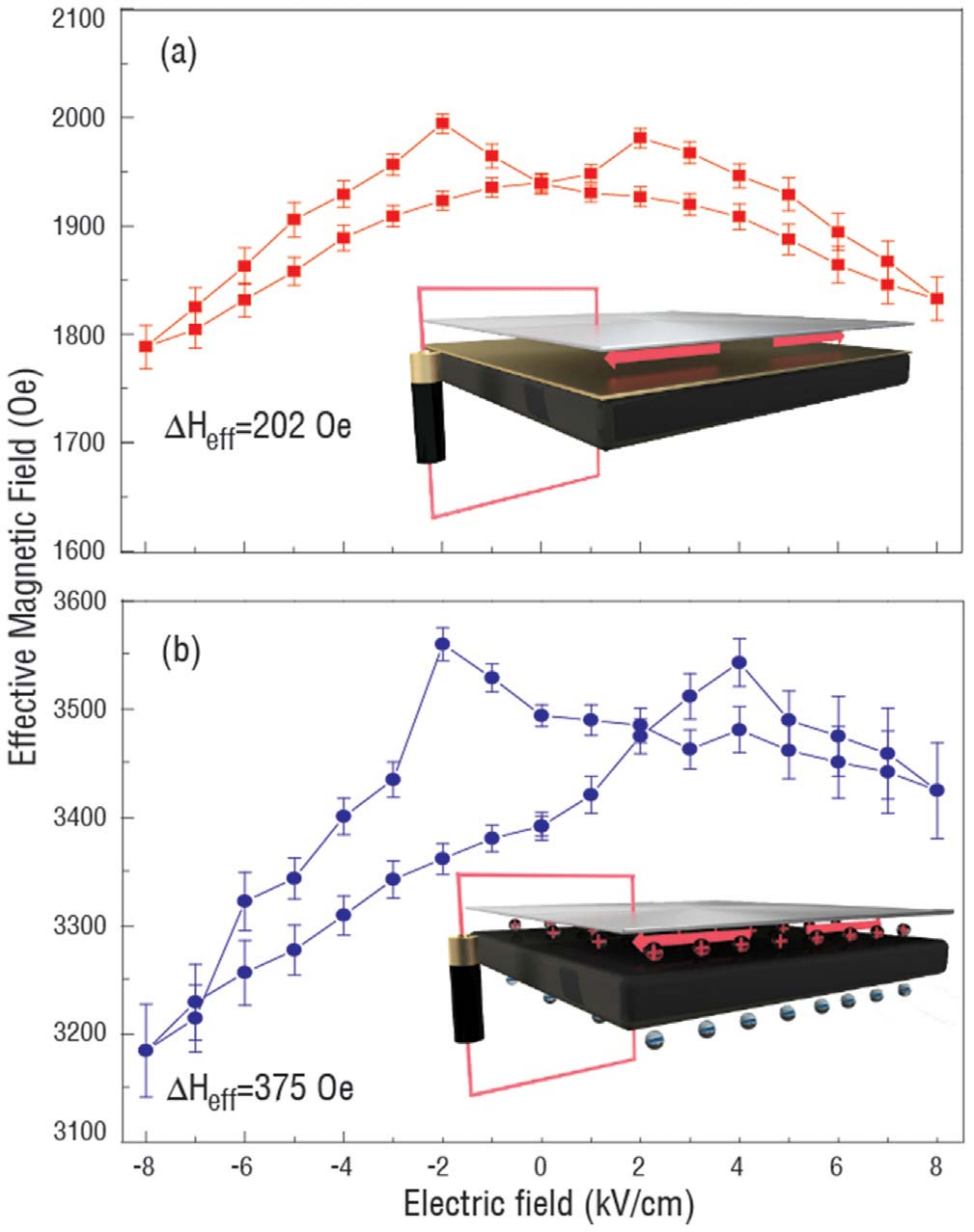
(a) FMR fields of NiFe/Cu/PMN-PT (011) and (b) NiFe/PMN-PT (011) upon applying different electric fields with the bias magnetic field applied along the in-plane [0–11] direction. Insets show schematic of NiFe/PMN-PT heterostructure (up) with strain and surface charge at the interface and NiFe/Cu/PMN-PT heterostructure with only strain at the interface (down).

As for NiFe/Cu/PMN-PT, the inserted Cu layer played the role of an isolation layer. In that case, no screening charge exists on the NiFe/PMN-PT interface. Thus pure strain mediated magnetoelectric coupling was controlled by the strain transferred through the Cu thin layer induced by the electric field on the PMN-PT due to the piezoelectric effect. As expected, a standard symmetric ‘Butterfly’ curve of the FMR effective magnetic field as the function of electric field was observed, as shown in Figure 2 (a), having a maximum *H_eff_* tunability of 202 Oe. This is corresponds to the symmetric strain *vs* E curve of PMN-PT (Figure 1S). In the FMR field sweeping mode, the magnetic field was applied along the in-plane [0–11] direction.

Interestingly for comparison, the NiFe/PMN-PT was measured in the FMR field sweeping mode under same experimental conditions. By removing the isolating Cu layer, a completely different magnetoelectric coupling phenomenon was observed as shown in Figure 2 (b). An asymmetric effective magnetic field as a function of electric field was observed. The maximum effective magnetic field shift was increased to ~375 Oe. The FMR effective magnetic field was increased due to the different surface anisotropy of NiFe/Cu and NiFe/PMNPT. More importantly, in NiFe/Cu/PMN-PT, the FMR magnetic effective field remained unchanged (~1940 Oe) at zero electric field due to the linear piezoelectric effect; while in NiFe/PMN-PT, two different magnetic effective fields (i.e., ~3390 Oe and ~3494 Oe, respectively) were induced depending on the electric polarization state of PMN-PT. The remnant polarization induced charge on NiFe/PMN-PT interface, thus modified the magnetic anisotropy via the screening charge effect. While in NiFe/Cu/PMN-PT, because of the conductive Cu layer (the charge screening length in Cu is ~0.6 A), the charge was unable to accumulate on the NiFe/Cu interfaces, resulting in non-existence of a charge mediated magnetoelectric effect. A simultaneous strain and charge mediated magnetoelectric coupling could possibly take place on NiFe/PMN-PT interface. In NiFe/PMN-PT heterostructure, two stable and reversible effective magnetic field states were established at zero electric field for achieving non-volatile switching of the resonance field by reversing electric field.

**Figure 3:**
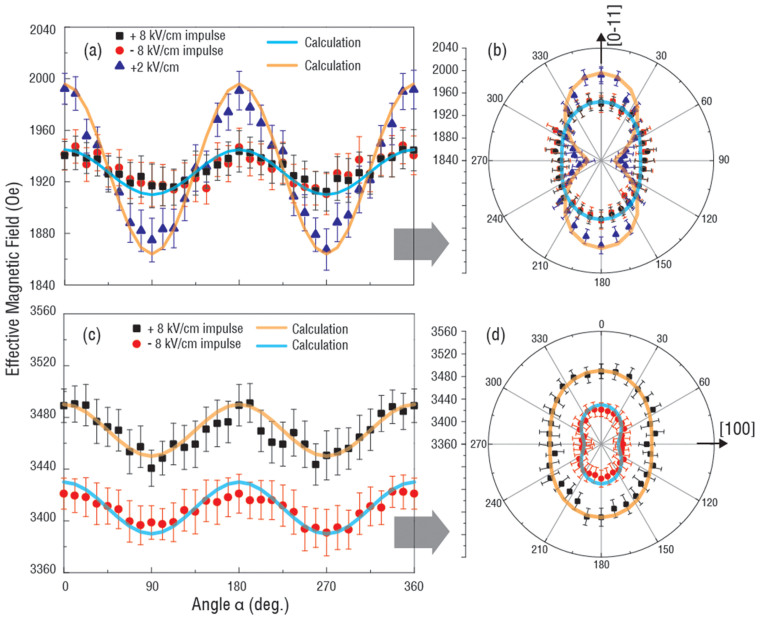
(a) Angular dependence of FMR effective magnetic fields of NiFe/Cu/PMN-PT and (c) NiFe/PMN-PT under different electric fields, where solid lines are the calculated curves. α is the angle between applied magnetic field and [0–11]direction. (b) and (d) show the polar graph transferred from (a) and (b), respectively.

In order to further investigate the distinct origin of magnetoelectric coupling effects between NiFe/PMN-PT and NiFe/Cu/PMN-PT, the angular dependence of FMR spectra were acquired with different applied electric fields. In FMR field sweeping mode, the magnetic field was first applied along in-plane [0–11] direction defined as 0° (360°) and the step of the increment was 15°. In NiFe/Cu/PMN-PT, with the applied electric field impulse of +8 kV/cm and −8 kV/cm (from 0 kV/cm through ± 8 kV/cm to 0 kV/cm), the FMR angular dependence depicts a uniaxial anisotropy with a magnetic easy axis along [100] direction, as shown in Figure 3(a) & (b). However due to the release of piezo-strain at zero electric field and the symmetric behavior of the piezoelectric “butterfly” curve (Figure S1), there was no magnetic anisotropy change. While applying an electric field of 2 kV/cm, the effective magnetic field increased along the [0–11] direction and decreased along the [100] direction. The in-plane magnetic anisotropy change is due to the linear piezoelectric effect of (011) oriented PMN-PT, in particular, the anisotropic in-plane piezoelectric coefficients of PMN-PT (*d_31_* = −1800 pC/N and *d_32_* = 900 pC/N). The change of the effective magnetic field in both directions was ~45 Oe which is consistent with the FMR field response in Figure 2 (a). A sharp contrast was observed in NiFe/PMN-PT as shown in Figure 3 (c) & (d) by measuring the angular dependent FMR spectra after applying +8 kV/cm and −8 kV/cm electric field impulses, respectively. The angular dependence FMR fields both exhibit the uniaxial anisotropy behavior; however, the +8 kV/cm electric field impulse induced a high effective magnetic field while −8 kV/cm induced a low effective magnetic field. This is a direct evidence of the existence of surface charge induced out-of-plane anisotropy change. The experimental data and calculated curves fit well, which will be discussed later. The change of the effective magnetic field was around 80 Oe which corresponds with Figure 2 (b) where there exists around a 100 Oe discrepancy of the FMR field at zero electric field. With the FMR spectra collected in the field sweeping mode, magnetic field along [0–11] direction, and an applied electric impulse of +8 kV/cm or −8 kV/cm, as showed in Figure S3, the NiFe film exhibited a FMR shift of around 90 Oe.

Recent work reported the influence of ferroelectric domain configuration on the magnetism of ferromagnetic layer in multiferroic heterostructure^33^,^34^,^35^,^36^. Liu et al.^33^ demonstrated a non-volatile switching of Verwey transition of Fe_3_O_4_ with the 71° ferroelastic domain wall switching. The unique ferroelastic pathway was achieved by sweeping the electric field near the electric coercive field of PMN-PT. In this experiment, the sweeping electric field upon the PMN-PT was much larger than its coercive field. In this configuration, most of the domain wall motions induced by electric field in PMN-PT were 180° ferroelectric domain wall switching. This would results in a “butterfly” like piezostrain vs electric field curve (Figure S1). Thus the non-volatile switching of effective magnetic field in NiFe/PMN-PT is not originated from the ferroelastic domain wall switching, but from the co-existence of charge and strain mediated magnetoelectric coupling. With the insertion of a 5 nm Cu layer, NiFe/Cu/PMN-PT showed a “butterfly” like effective magnetic field vs electric field curve [Figure 2(a)].

**Figure 4:**
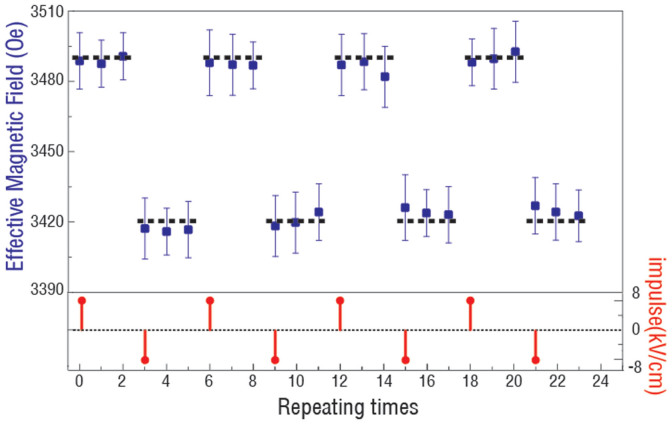
Electric field impulse induced non-volatile switching of FMR field of NiFe/PMN-PT with a magnetic field applied along [0–11] direction.

Figure 4 shows a voltage-impulse-induced FMR field tuning in NiFe/PMN-PT (011) with a magnetic field along [0–11] direction, which demonstrates a robust and repeatable non-volatile switching of effective magnetic field.

## Discussion

In order to understand the co-existence of strain and charge mediated magnetoelectric coupling, we calculated the angular dependence of the FMR field. Figure S4 shows the schematic of the established coordinate system in NiFe/PMN-PT (011). The magnetization of NiFe has a *θ* degree angle along Y axis, an in-plane (X-Y plane) projection with *φ* angle along X axis (easy axis), and a magnetic field *H* at *α* angle from easy axis. In NiFe/PMN-PT, the total magnetic energy of NiFe thin film can be expressed as: 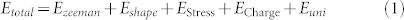
where *E_zeeman_* is the Zeeman energy, *E_shape_* the demagnetizing energy, *E_surf_* the surface anisotropy energy, *E_Stress_* the magnetoelastic energy, and *E_uni_* the uniaxial anisotropy. *E_total_* can be further expressed as, 
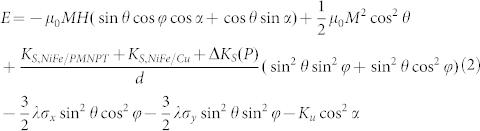
where *K_S, NiFe/PMNPT_* and *K_S, NiFe/Cu_* are magnetic surface anisotropy energy between NiFe and PMNPT and the Cu layer, respectively; *ΔK_s_(P)* denotes the change of surface anisotropy energy under electric field induced electric polarization; *σ_x_* and *σ_y_* are strain along X and Y axis of NiFe film, respectively; *M*, *d* and *λ* are the magnetization, thickness and magnetostrictive constant of the NiFe thin film, respectively. We can solve for the total energy into the FMR equation below by minimizing the energy equation. 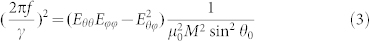
where *f*, *γ* and *μ_0_* are resonance frequency, gyromagnetic ratio of 2.8 MHz/Oe and permeability of free space, respectively.

Thus the solved FMR equation can be expressed as, 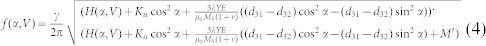

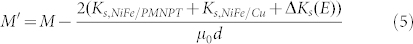
where *Y* and *ν* are the Young's modulus and Poisson ratio of PMN-PT; d_31_ and d_32_ are the in-plane piezoelectric coefficients of PMN-PT; *H (α, V)* denotes the effective magnetic field which is determined by the electric field *V* and the angle *α*.

Given a fixed frequency *f*, the change in the effective magnetic field induced by a piezo-strain and screening charge on the PMN-PT interface can be solved as, 
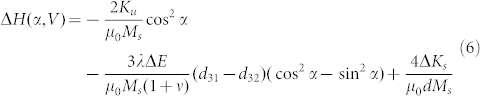
As a comparison, the change of the effective magnetic field in NiFe/Cu/PMN-PT was also calculated. Because of the isolation created by the thin Cu layer, no screening charge can accumulate on the NiFe layer, thus *ΔK_s_(V)* = 0, and Equation (5) should be re-written as, 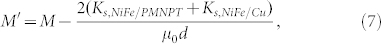
then the change of the effective magnetic field of NiFe/Cu/PMN-PT is expressed as, 
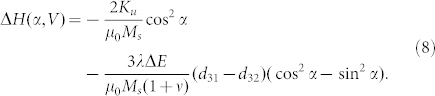
Clearly, from Equation (6), the second term of the equation, the magnetoelastic induced effective magnetic field is zero at zero electric field, which leads to a uniaxial angular dependence of the effective magnetic field. The change of the effective magnetic field is dependent on the change of magnetic surface anisotropy or the remnant polarization of the ferroelectric PMN-PT, which is consistent with Figure 2 (b) In NiFe/Cu/PMN-PT, from Equation (8) the pure strain induced effective magnetic field shows a strong dependence on α, thus a dipole like FMR field angular dependence curve is shown in Figure 3 (b). Since the inserted Cu layer between the NiFe and PMN-PT has a very small thickness of 5 nm, we ignore the slight reduction of piezo-strain transferred to NiFe due to the Cu layer (see Figure S5), and the small difference on magnetic moment of NiFe/PMN-PT and NiFe/Cu/PMN-PT (see Figure S2). By subtracting Equation (8) from Equation (6), the pure charge induced change of effective magnetic field can be obtained as, 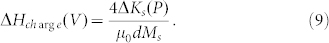
This effective magnetic field corresponds with Figure 2 (b). By taking account of Equations (6) and (8), the calculated curve in Figure 3 fits well with the experimental data for the angular dependence of the FMR field.

**Figure 5:**
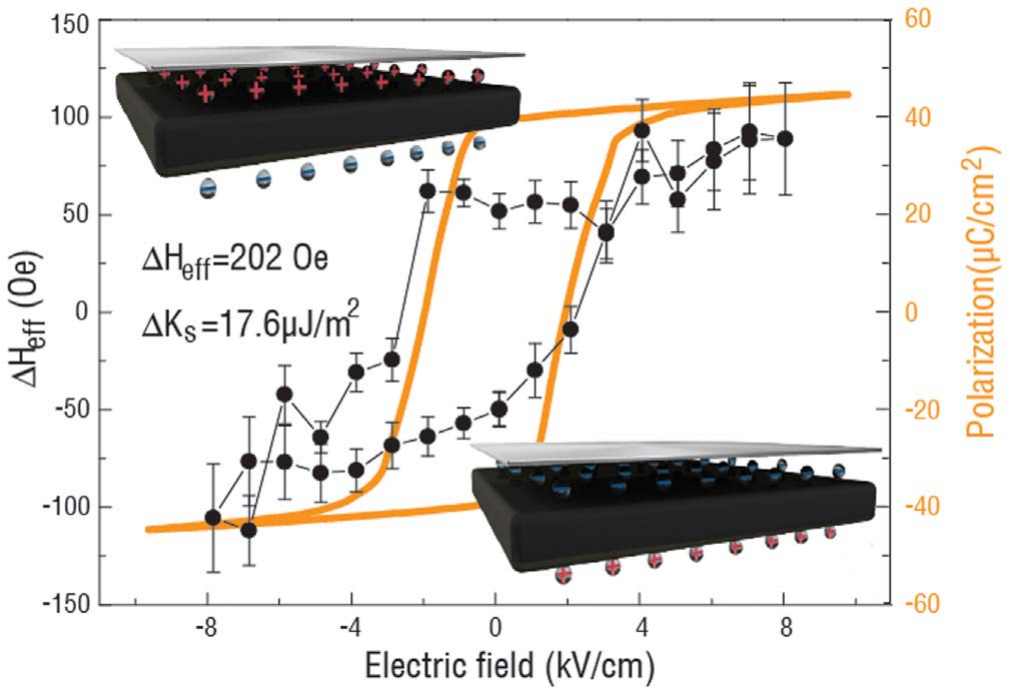
The change of the effective magnetic field upon the applied electric field, induced by pure screening charge effect in NiFe/PMN-PT (black) and P(E) loop of PMN-PT(orange). Insets show the schematics of the positive (up) and negative (down) screen charge on the NiFe interface.

Experimentally, the change of the effective magnetic field of NiFe/Cu/PMN-PT and NiFe/PMN-PT can be obtained from Figure 2 (a) and (b), then by subtraction, the *ΔH_charge_* as a function of electric field is obtained shown in Figure 5.

The loop-like effective magnetic field shares a similar shape with the electric polarization of PMN-PT as a function of electric field (orange line) which has a sudden jump at the electric coercive field of around ± 2 kV/cm. The links between the ΔH-E and P-E loops demonstrate the control of the effective magnetic field by electric field via the screening charge mechanism. The hysteretic ΔH-E loop reveals how the magnetic anisotropy of ferromagnetic materials would respond to the surface charge, which is proportional to the ferroelectric polarization as a function of electric field. Of interest, the total change of the effective magnetic field by pure charge effect of around 202 Oe is almost equal to the total change of the effective magnetic field by pure strain effect derived from Figure 2 (a) in NiFe/PMN-PT with the ultra-thin NiFe film of 1 nm. With the total change of the effective magnetic field of 202 Oe, the total *ΔK_s_* induced by the screening charge can be calculated using Equation (9) as 17.6 μJ/m^2^.

## Conclusions

In summary, we have demonstrated a reversible and non-volatile switching of magnetism by electric field via combined charge and strain mediated magnetoelectric coupling in an ultra-thin NiFe and PMN-PT multiferroic heterostructure. Voltage controlled ferromagnetic resonance of the heterostructures was utilized for quantitatively studying the magnetoelectric behavior. The change of effective magnetic field of 375 Oe was observed in NiFe/PMN-PT heterostructure with the strain and surface charge mediated magnetoelectric coupling. By subtracting the strain induced effective magnetic field, we obtained the natural response of magnetization of ultra-thin NiFe to screening charge, which displays a similar trend as polarization of PMN-PT versus electric field. Due to the different remnant polarization state of PMN-PT the non-volatile behavior was observed in NiFe/PMN-PT heterostructure with an effective magnetic field change of 104 Oe at zero electric field. The co-existence of strain and charge mediated magnetoelectric coupling in ultra-thin magnetic/ferroelectric heterostructures could lead to non-volatile magnetoelectric devices with significantly enhanced magnetoelectric coupling.

## Method

Cu (5 nm)/NiFe (1 nm)/Cu(5 nm) and Cu/NiFe (1 nm) were deposited by DC magnetron sputtering on (011) oriented single crystalline PMN-PT substrates at room temperature. The base pressure of the chamber was below 1 × 10^−7^ Torr. The thickness of the deposited thin films was controlled carefully by the deposition time with a fixed DC power of 50 W. The ferroelectric property of PMN-PT was measured by the Radiant Ferroelectric characterization system. The strain *vs* E curve was measured using a photonic sensor by sweeping the sinusoidal electric field with the amplitude of 9 kV/cm. The magnetic hysteresis loops were measured using a superconducting quantum interference device (SQUID) magnetometer at room temperature. The ferromagnetic resonance spectra were measured using our X-band ESR test system at room temperature in field sweeping mode. The microwave frequency was fixed at 9.5 GHz and power was −20 dBm. In the FMR field angular dependence measurements, the samples were fixed on the sample holder with a precise angle rotator. All electric field impulses used in the experiments were controlled by an electric relay and the duration of the impulses was 100 ms.

## Author Contributions

T.N. and Z.Z. have equally contribution to this work with preparation and characterization of the samples. N.S. and T.N. initiated the original idea and T N. wrote the main manuscript. T.N. and Z.Z. did the thin film deposition process and the main experiments. M.L., X.Y., Y.G., H.L., S.V., X.W., J.C., S.A., E.H., R.R., K.K. and S.S. assist the FMR experiments. B.A.A. and Don Heiman measured the M (H) loop of NiFe/PMN-PT heterostructure using SQUID. H.L., B.M.H. and G.J.B. helped on the characterization of the piezoelectric substrate PMN-PT.

## Supplementary Material

Supplementary Information Supplementary Information
