# Supplementary material for: Quantification of strain and charge co-mediated magnetoelectric coupling on ultra-thin Permalloy/PMN-PT interface
Source: Sci Rep. 2014 Jan 14;4:3688. doi: 10.1038/srep03688 (PMC3891213; doi:10.1038/srep03688)
Supplement: Supplementary Information [file srep03688-s1.doc]

**Supplementary Information**

*Tianxiang Nan*^1^, Ziyao Zhou*^1^, Ming Liu^2^, Xi Yang^1^, Yuan Gao^1^,* [*Badih A. Assaf*](http://arxiv.org/find/cond-mat/1/au:+Assaf_B/0/1/0/all/0/1) *^3^, Hwaider Lin^1^, Siddharth Velu^1^, Xinjun Wang^1^, Haosu Luo^4^, Jimmy Chen^5^,* *Saad Akhtar,^6^ Edward Hu,^7^ Rohit Rajiv,^8^ Kavin Krishnan,^9^ Shalini Sreedhar,^10^* [*Don Heiman*](http://arxiv.org/find/cond-mat/1/au:+Heiman_D/0/1/0/all/0/1)*^3^, Brandon M. Howe^2^, Gail J. Brown^2^, and Nian X. Sun*^#1^

*These authors contribute equally.

# Correspondence and requests for materials should be addressed to Nian Sun: [n.sun@neu.edu](mailto:n.sun@neu.edu)

^1^. Department of Electrical and Computer Engineering, Northeastern University, Boston, MA, USA

^2^. Materials and Manufacturing Directorate, Air Force Research Laboratory, Wright-Patterson AFB, OH, USA

^3^. Department of Physics, Northeastern University, Boston, MA

^4^. Shanghai Institute of Ceramics, Chinese Academy of Sciences, Shanghai, China

^5^. Winchester High School, Winchester, MA

^6^. Foxborough High School, Foxborough, MA

^7^. Boston Latin School, Boston, MA

^8^. Phillips Exeter Academy, Exeter, NH

^9^. Advanced Math & Science Academy Charter School, Marlborough MA

^10^. Weston High School, Weston, MA


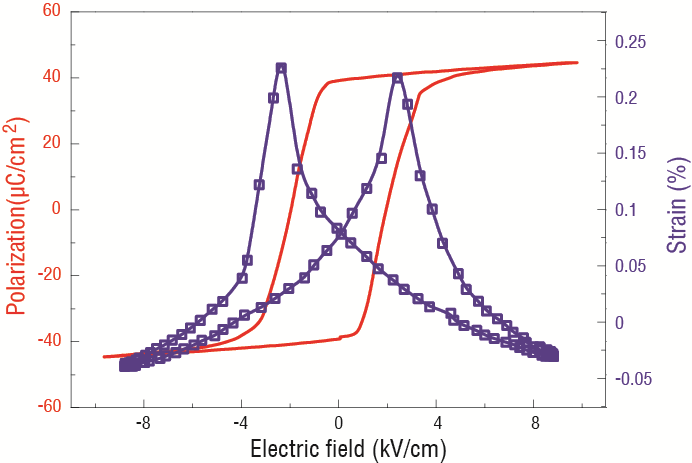


Figure S1 Polarization (red) and piezoelectric strain (blue) of (011) oriented PMN-PT as a function of electric field, where the electric coercive field was found at ±2 kV/cm.


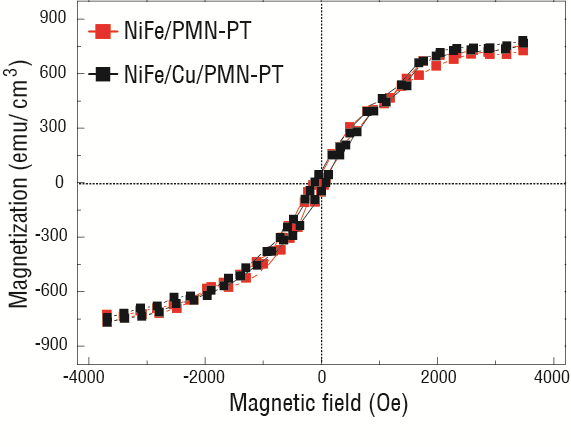


Figure S2 Magnetic hysteresis loop of NiFe/PMN-PT (red) and NiFe/Cu/PMN-PT (black) heterostructures measured by SQUID magnetometer, with a saturation magnetization of 875 emu cm^-3^. There’s very slight difference on the magnetic moment between NiFe/PMN-PT and NiFe/Cu/PMN-PT heterostructures. We assume they have the same moment in the calculation.


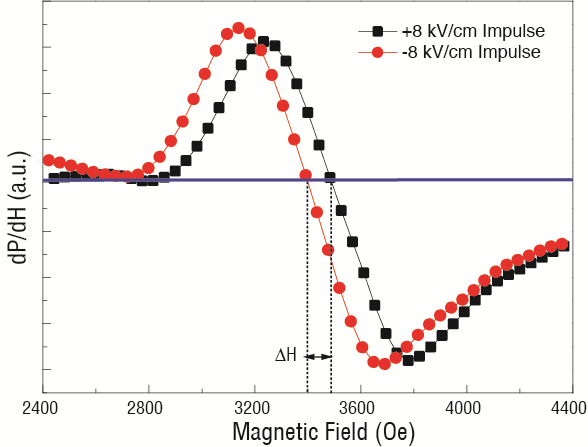


Figure S3 FMR spectra in field sweeping mode at 9.5GHz of NiFe/ PMN-PT with applied electric field impulse of +8 kV/cm (black) and -8 kV/cm (red). The change of the resonance field was around 80 Oe.


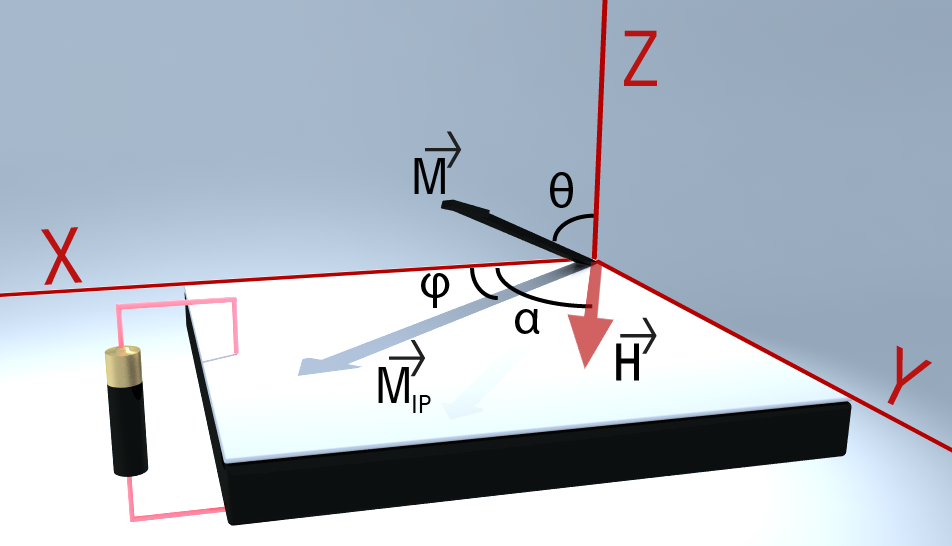


Figure S4 Schematic of NiFe/ PMN-PT in coordinate system. The magnetization of NiFe has θ angle along Y axis and in-plane (X-Y plane) projection with ϕ angle along X axis (easy axis), magnetic field H at α angle from easy axis.


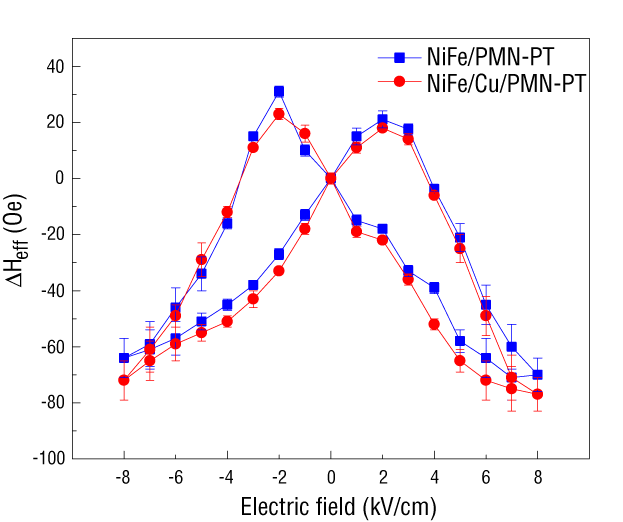


Figure S5 The change of effective magnetic field upon the applied electric field in NiFe(10nm)/Cu(5nm)/PMN-PT and NiFe(10nm)/PMN-PT. In both NiFe(10nm)/Cu(5nm)/PMN-PT and NiFe(10nm)/PMN-PT samples, they have similar trend and shape of the change of effective magnetic field as a function of the electric field. The change of induced magnetic field in the two cases is nearly equal to each other. The very small discrepancy may due to the error of the measurements. The total change of induced magnetic field ~90 Oe in both cases is smaller than the induced magnetic field in NiFe(1nm)/PMN-PT, which is resulted from the decrease of the magnetostriction of NiFe with the increase of the thickness of NiFe (Y. K. Kim, Appl. Phys. Lett. 68, 13 1996). The influence of the insertion Cu(5nm) layer on the intensity of the strain-mediated magnetoelectric coupling is very small.
